# Protein-Repellence PES Membranes Using Bio-grafting of Ortho-aminophenol

**DOI:** 10.3390/polym8080306

**Published:** 2016-08-15

**Authors:** Norhan Nady, Ahmed H. El-Shazly, Hesham M. A. Soliman, Sherif H. Kandil

**Affiliations:** 1 Polymeric Research Department, Advanced Technology and New Materials Research Institute (ATNMRI), City of Scientific Research and Technological Applications (SRTA-City), New Boarg El-Arab City 21934, Alexandria, Egypt; 2 Chemical and Petrochemical Engineering Department, School of Energy, Environmental and Chemical and Petrochemical Engineering, Egypt-Japan University of Science and Technology (E-JUST), New Boarg El-Arab City 21934, Alexandria, Egypt; elshazly_a@yahoo.com; 3 Nanomaterials Research Department, Advanced Technology and New Materials Research Institute (ATNMRI), City of Scientific Research and Technological Applications (SRTA-City), New Boarg El-Arab City 21934, Alexandria, Egypt; h.soliman@mucsat.sci.eg; 4 Department of Materials Science, Institute of Graduate Studies and Research, Alexandria University, Alexandria 21544, Egypt; s.kandil@usa.net

**Keywords:** protein-repellence, poly(ethersulfone), green surface modification, bio-catalysis, ortho-aminophenol

## Abstract

Surface modification becomes an effective tool for improvement of both flux and selectivity of membrane by reducing the adsorption of the components of the fluid used onto its surface. A successful green modification of poly(ethersulfone) (PES) membranes using ortho-aminophenol (2-AP) modifier and laccase enzyme biocatalyst under very flexible conditions is presented in this paper. The modified PES membranes were evaluated using many techniques including total color change, pure water flux, and protein repellence that were related to the gravimetric grafting yield. In addition, static water contact angle on laminated PES layers were determined. Blank and modified commercial membranes (surface and cross-section) and laminated PES layers (surface) were imaged by scanning electron microscope (SEM) and scanning probe microscope (SPM) to illustrate the formed modifying poly(2-aminophenol) layer(s). This green modification resulted in an improvement of both membrane flux and protein repellence, up to 15.4% and 81.27%, respectively, relative to the blank membrane.

## 1. Introduction

Membrane fouling can be defined as the irreversible adsorption/deposition of components on and into the surface of membrane. Reversible fouling can be easily removed by backwashing (reverse the direction of the rinsing fluid) of the membrane; thus, reversible fouling cannot be considered as great a problem as irreversible fouling. Fouling depends mainly on the physicochemical interactions between the membrane material and the components in the flowing fluid. The properties of both the components and the membrane material are of importance, because they determine whether these components can attach/adsorb onto the membrane surface [1,2]. Membrane fouling can be caused by various components, such as colloidal particles [3,4], minerals [5,6], antifoam [7,8], proteins [9,10], and microorganisms [11,12]. Various remediation methods have been proposed to influence the interaction between these foulants (i.e., the components that can be adsorbed on or onto the membrane surface) and membranes, including adjustment of the system hydrodynamics [13,14,15], surface modification [16,17], and regular cleaning [18,19]. Regular cleaning can be done physically (i.e., backwashing or mechanical vibration) [17,18] or chemically (i.e., flowing acidic, alkalin, cleaning agent, and/or surfactant) [19]. Practically, in-depth fouling is very hard to remove because the foulant will also partially block the local flow that is needed to remove and carry away the foulant. Clearly and even more relevantly, once a foulant is attached to the membrane, it works as an initiator for attachment of more foulants. For example, protein adsorption can be an initial step for attachment and growth of microorganisms (i.e., biofilm formation) [20].

The hydrophobicity of the membrane surface has been stated as the reason of membrane fouling. Thus, increasing the hydrophilicity of different hydrophobic membrane materials has been reported using surface modification methods, including coating, composite, blending, chemical, plasma and UV-assistant grafting, or a combination of two or more methods [16,17]. The effectiveness of increasing the hydrophilicity of the membrane surface for the minimize adsorption of foulants such as protein has been explained as depending on the fact that the hydrophilic surface attracts so much water that adsorption of proteins is reduced [17,21,22]. However, surface structure has significant impact on membrane anti-fouling performance [23]. In this respect, both steric hindrance and the osmotic effect of hydrated (grafted) polymer brushes contribute to resistance against membrane fouling [24]. Besides, novel low-fouling surfaces or membranes with a high regular ‘‘patterns’’ have been investigated [25]. Although the effect of the membrane roughness on its tendency for foulant adsorption is debated [17], the change in the surface topography by applying such regular patterns has been shown to enhance its repellence for different foulants [26,27]. The definite explanation of the effect of these novel surface patterns is not completely understood yet. Meanwhile, the interplay by different influencing parameters such as surface energy, interfacial mass transfer, the hydrodynamic interactions, etc. has been proposed to induce localized turbulence and/or shear stresses that help in reduction the foulant adsorption [28,29,30,31].

**Figure 1:**
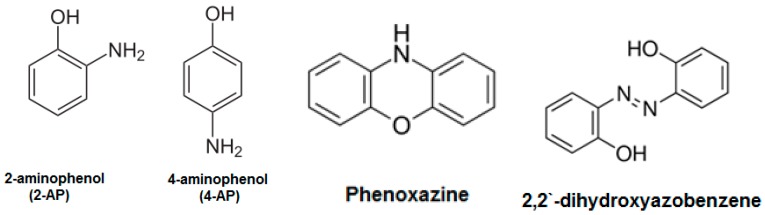
Molecular structure of 2-aminophenol, 4-aminophenol, phenoxazine, and 2,2′-dihydroxyazobenzene.

The position of side groups (i.e., amino and hydroxyl) in aminophenol strongly affect on its polymerization and on the structure of the produced oligomers or polymers of poly(aminophenol) (see Figure 1) [32,33]. The chemical and electrochemical polymerization of the three isomers of aminophenol have been presented [32]. The electrochemical oxidation of 4-aminophenol (4-AP) on different materials of electrode has been published [34,35,36,37]. In these studies, 4-benzoquinone was the proposed product of the electrooxidation of the 4-AP in aqueous medium [23,24,25]. In addition, the effect of chemically polymerized 4-AP on inhibition the growth of different bacterial strains such as *Staphylococcus aureus* (gram positive) and *Escherichia coli* (gram negative) at different levels of the polymer concentration has been reported [37]. In addition, the enzymatic-catalyzed polymerization of 4-AP onto poly(ethersulfone) (PES) membranes has been reported to significantly improve the protein repellence of the modified membranes [38], whereas the chemical and electrochemical polymerization of 3-aminophenol (3-AP) takes place via C–O and/or C–N coupling [32,39]. The electrochemical polymerization of 3-AP in aqueous solution on SnO_2_ electrodes proposed that only the amino group was oxidized while the hydroxyl group remained unchanged [39]. However, the oxidation of the 3-AP on a platinum electrode in acid medium yielded a polymeric product that may have a crosslinked or a linear structure analogous to polyphenol [32]. Meanwhile, the structure of the electrochemical polymerization of 2-aminophenol (2-AP) has been investigated by different researchers [40,41,42]. Most studies proposed that the formed oligomers or polymers of poly(2-AP) have a ladder structure with a repeating phenozaxine units [40,41]. In addition, a linear dimer formed by N–N coupling; 2,2′-dihydroxyazobenzene, has been identified as a polymerization product of 2-AP in non-acidic media [42].

**Figure 2:**
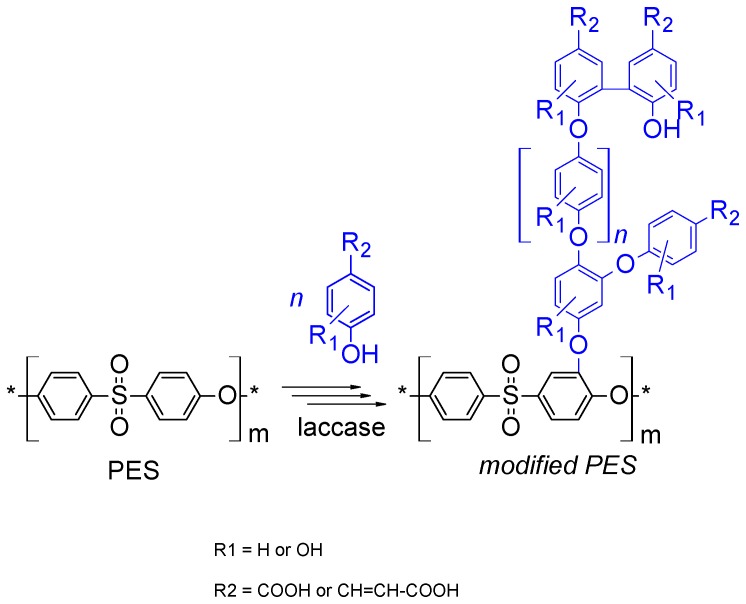
Tentative mechanism for the reaction of laccase-generated radicals with poly(ethersulfone) (PES), and subsequent formation of grafted brushes [50].

Poly(arysulfone) membranes such as poly(ethersulfone) (PES) have a high ability to interact with different molecules such as organic substances, proteins, live cells, etc. that result in membrane fouling. To reduce (or eliminate) this fouling, several surface modification methods have been reported [43,44]. In previous work [45,46,47], the modification of PES membranes using laccase biocatalyst and phenolic acids modifiers has been investigated [48]. The used laccase enzyme has ability to oxidize the phenolic acids [48,49] to their free radical forms. The formed reactive radicals can covalently react to form homopolymer and/or bind onto PES membrane, mostly through their phenolic OH-groups, as shown in the proposed layer structure in Figure 2 [50]. Furthermore, this laccase-catalyzed modification has been used to modify the PES membrane using 4-aminophenol isomer and induces significant reduction in protein adsorption [38].

In this paper, 2-AP isomer modifier was used (i.e., monomer to be grafted onto the PES membrane) and enzyme laccase biocatalyst to modify the PES membrane; 5 and 15 mM 2-AP modifier and 0.5 U·mL^−1^ laccase biocatalyst were used to modify the PES membranes at 30, 60, and 120 min reaction (modification) times; sodium acetate buffer (0.1 M of pH 5.5) was used; and blank and modified commercial membranes were characterized using the following techniques: the membrane total color change, the added modifier per unit area (the grafting yield), pure water flux, static water contact angle on laminated PES layer on silica slides (silicon dioxide layer on silicon support), reduction in irreversible protein adsorption, and flux reduction due to the irreversible protein adsorption. A scanning electron microscope (SEM) was used to image both the surface and the cross-section of the commercial blank (unmodified) and modified membranes and also to image the surface of both the blank and the modified laminated PES layer. A scanning probe microscope (SPM) was used to illustrate the shape of the formed modifying layer on the laminated PES layer. The general outlook of the obtained data allowed us to conclude the effect of bio-catalyzed grafting of PES membranes (or surfaces) by 2-AP modifier on antifouling property of PES membrane or surface. Part of this work has been presented at the 2014 World congress on Advances in Civil, Environmental and Materials Research (ACEM14), Busan, Korea [51].

## 2. Materials and Methods

### 2.1. Materials

The following materials were purchased from Sigma-Aldrich (Saint Louis, MO, USA): Ortho-aminophenol (2-AP, ≥99.5%), dichloromethane ACS (DCM, 99.9%), catechol (>97%), sodium acetate (≥99%), and acetic acid (99.9%). Poly(ethersulfone) (PES) polymer was gifted from BASF (Ludwigshafen, Germany). One hundred fifty-millimeter silicon wafers with 2.5 nm native oxide top layer were obtained from wafer Net Inc. (San Jose, CA, USA). Commercial PES membranes were purchased from Sartorius (Göttingen, Germany) (0.2 µm pore size, symmetric, 50 mm diameter, 150 µm thickness, > 28 mL·min^−1^·cm^−2^ water flow rate at ∆P = 1 bar). Laccase (>10.4 U·mg^−1^) from *Trametes versicolor* was obtained from Fluka (Gillingham, England). MilliQ water was used to prepare fresh solutions before each experiment.

### 2.2. Enzyme Activity (Assay)

The activity of laccase enzyme has been determined as was described in previous work [52] using catechol [53]. The amount of enzyme required to oxidize 1 µmol of catechol per min at 25 ± 1 °C was used as a unit of laccase activity.

### 2.3. Model Membrane Preparation

Silicon strips coated by a thin layer of silicon dioxide were used as support of pure PES layers (surfaces) as was described in previous work with slight modification [50]. The square (1 × 1 cm^2^) silicon dioxide strips were used to laminate PES layer on it using spin-coating of 0.5% *w/w* polymer solution (PES) in DCM solvent. Coating time and speed were 30 s and 2500 rpm, respectively.

### 2.4. Membrane (Surface) Modification

The membranes were laid on the bottom of glass beakers including 40 mL sodium acetate buffer (0.1 M) containing both the 2-AP modifier and the laccase enzyme. Air was bubbled (as O_2_ source and for gentle mixing) inside the reaction medium. Reaction times of 30, 60, or 120 min (modification) were used. The membranes were washed by strong spray flushing of deionized water, followed by dipping and shacking in freshly boiled deionized water for three times. The modified membranes were kept in desiccators for 48 h before using.

### 2.5. Membrane Color Change

The CIELAB coordinates for the modified real membranes were measured with SP62 Sphere Spectrophotometer (Lansing, MI, USA) as was described in previous work [50,51,52,53,54,55]. ∆E* (membrane total color change) was determined relative to the blank commercial membrane that was used as a standard (compare mode was used). The smallest aperture size (8 mm diameter) was used. The average of three readings, which were taken from three different places on each sample, was calculated. The membranes were dried for more than 48 h before the membrane color change was determined.

### 2.6. Pure Water Flux

Water flux was measured as was described in previous work [48] using a Millipore stirred ultrafiltration cell with 13.4 cm^2^ active transport area. Dead-end operation mode was used.

### 2.7. The Added Modifier per Unit Membrane Surface Area (Grafting Yield)

The added modifier was calculated gravimetrically from the difference in weight of the membrane before and after modification and was related to the unit area of membrane surface as was described in previous work [49,50]. The membranes were kept for 48 h in glass-covered dishes in desiccator to remove any moisture.

### 2.8. Protein Repellence

The adsorption of Bovine serum Albumin (BSA) on the blank and the modified commercial membranes was evaluated as was described in previous research [49,50]. Briefly, the membranes were shaken in 50 mL BSA solution (1 g·L^−1^) at 25 °C for 24 h. Adsorbed BSA was determined from the difference between the original BSA concentration and the residual BSA concentration in the shaken solution using the pre-prepared standard curve and UV-spectrophotometer (Kyoto, Japan) at 280 nm wavelength.

### 2.9. Scanning Electron Microscope (SEM)

The surface and the cross-section of both blank and modified commercial membranes were scanned using a Jeol Jsm 6360LA Scanning Electron Microscope (SEM, Tokyo, Japan) as was described in previous work [49]. For cross-section imaging, the membranes were frozen by immersion in liquid nitrogen and then were fractured and were coated by Au. Magnifications of 5000× and 15,000× were used. The laminated PES layers or surfaces were imaged at a voltage of 10–20 KV after coating with Au. Magnifications of 15,000× and 30,000× were used.

### 2.10. Scanning Probe Microscope (SPM)

Blank and modified laminated PES layers were prepared on square strips (1 × 1 cm^2^) and were imaged using Scanning Probe Microscope (SPM, Shimadzu, Kyoto, Japan). Non-contact mode was used to image 5 × 5 µm^2^ strips.

### 2.11. Static Water Contact Angle

The static water contact angle on the blank and the modified laminated PES layers was determined using a Krüss DSA 100 apparatus (Hamburg, Germany). Drops of demineralized water (7 µL) were deposited on different spots of each laminated PES layer. Four different spots on each layer of two different independently modified slides were measured and were averaged. All the tested membranes were kept in desiccators for 48 h before measuring. MilliQ water was used in all the experiments.

### 2.12. Membrane Tensile Strength

Samples were cut in a dumbbell shape with dimensions as was described in previous work [49]. Constant crosshead speed of 6 mm/min was used. Two samples from each membrane were tested.

## 3. Results and Discussion

**Figure 3:**
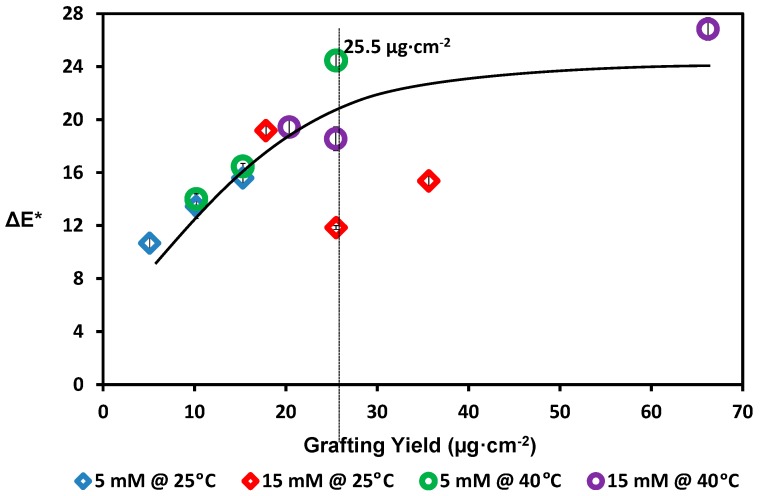
Change of the membrane color (∆E*) with grafting yield; common reaction condition is pH 5.5 (0.1 M sodium acetate buffer), and 0.5 U·mL^−1^ enzyme (laccase). Reaction at 25 °C using 5 mM 2-AP (blue diamond) and 15 mM 2-AP (red diamond), and reaction at 40 °C using 5 mM 2-AP (green circle) and 15 mM 2-AP (purple circle). Resection times 30, 60, and 120 min were tested and the grafting yield was increased with increasing the reaction time. The solid black line is a guide for general trend.

In this study, 2-AP monomer was used to modify PES membranes using laccase-catalyzed grafting method. The laccase was used to catalyze conversion of the modifier 2-AP into its free radical form that is required for the grafting onto the PES membrane. The formed free radicals can react with each other to form homopolymers of poly(2-AP) and/or to graft on/onto membrane surface [49]. The modified membranes were intensively washed using boiled water until no absorption was determined by spectrophotometer. Figure 3 shows the effect of the grafting yield on the total color change of the membrane (∆E*). Different modification conditions were used as following: 5 and 15 mM 2-AP modifier concentrations, 25 and 40 °C reaction (modification) temperatures, and 30, 60, and 120 min reaction (modification) times. The concentration of enzyme was 0.5 U·mL^−1^ dissolved in 0.1 M sodium acetate buffer, which was used to keep the pH of the reaction medium at 5.5. As seen in Figure 3, the total color change of the membrane (∆E*) was increased gradually with the increases of the grafting yield up to 66.21 µg·cm^−2^ (the total color change of the membrane was 26.2 when using 15 mM 2-AP, 40 °C reaction temperature, and 120 min reaction time). The increases of each studied parameter e.g., modifier concentration, reaction time, etc. resulted in an increase in the grafting yield. Similar to the previous study using 4-AP modifier [38], different changes of the membrane color at 25.5 µg·cm^−2^ grating yield were determined (At 40 °C: (a) 5 and (b) 15 mM 2-AP modifier with 120 and 60 min reaction times, respectively; also at 25 °C: (c)15 mM 2-AP modifier at 60 min reaction time). However, the case of using 2-AP modifier differs from the case of using 4-AP modifier [38] regarding protein repellence. The three modified membranes using 2-AP modifier did not show as good a protein repellence as those using 4-AP modifier. Previous studies [17,39] emphasize the effect of both surface hydrophilicity and structure/shape of the formed modifying layer on the foulant repellence of the modified surface [49]. Regarding the static water contact angle as an indicator for the surface hydrophilicity, these three modified membranes using 2-AP modifier have not shown a noticeable change in the static water contact angle values, around 77° ((a) 76.4° ± 1.5°, (b) 74.1° ± 1°, and (c) 80.4° ± 3°, respectively; the blank PES surface has 75.9° ± 2° static water contact angle). Regarding the shape of the modified surfaces, (as shown in SEM images in the following discussion), the produced modifying surfaces are similar in shape and may also be similar in structure; more investigation on the produced structure are on going, whereas the membrane color increased more strongly with modification at 40 °C, most probably due to formation of dense layers that are more saturated in color [56] (i.e., have higher extinction coefficients). Color saturation (∆C*, not measured in this study) is a characteristic parameter indicating the intensity of the color: the color with a high saturation will appear more intense than the same color with less saturation. It seems that the three modification conditions produce the same grafting yield, static water contact angle, and the same shape and/or structure because they show almost the same protein repellence (i.e., the error limits have been considered) (see irreversible protein adsorption evaluation results in the following discussion). However, the total change in the color of the three membranes may be a result of change in their color saturation [54,56]. In addition, at grafting yield equal or greater than 25 µg·cm^−2^, there is no clear relation between the grafting yield and the total color change (∆E*) of the membranes. This may be interpreted by the fact that the increasing of the added modifier is accompanied with a difference in the color saturation, as previously illustrated (color depth, not measured in this work) [57,58,59], instead of the total color change of membranes. This may be attributed to the formation of extra layers instead of the increases in the grafting density (increase the length of the grafted oligomers rather than increases the number of the grafted oligomer) [56,57,58,59]. It is very clear that most of the tested membranes carry not more than 25.5 µg·cm^−2^ grafting yield (about 2.55 mg·m^−2^). Assuming the mass of one molecule of 2-AP is 18.1 × 10^−19^ mg and occupies about 1 nm^2^ of space, a dense monolayer would thus approximately yield a coverage of 1.8 mg·m^−2^. If we assume formation of phenoxazine [40,41], a dense monolayer would thus approximately yield a coverage of 3 mg·m^−2^. This is in full agreement with assuming the increasing in the grafting yield more than 25.5 µg·cm^−2^ will result in increasing the length of the grafted oligomers and/or adsorption rather than increasing the number of the grafted oligomer per unit area (grafting density) [59]. In addition, this rough calculations propose formation of one or two layers of modifying layer on the membrane as maximum, which can be the reason for avoiding the harmful effect on the membranes’ pores and consequently on the membranes’ original flux.

**Figure 4:**
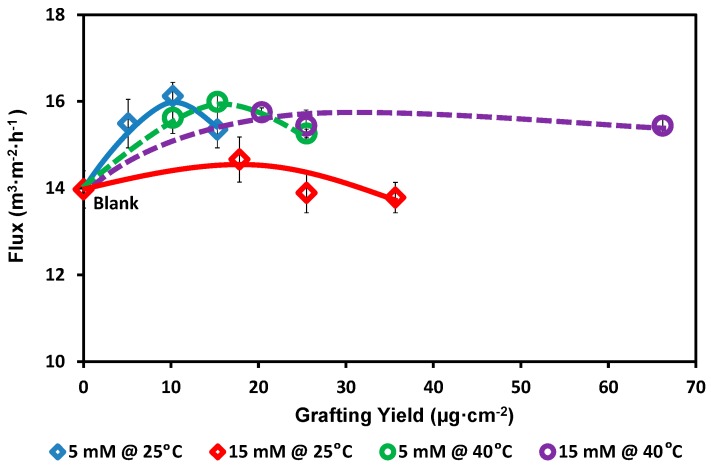
The effect of the grafting yield on the pure water flux of the membrane. Common reaction condition is pH 5.5 (0.1 M sodium acetate buffer), and 0.5 U·mL^−1^ enzyme (laccase). Reaction at 25 °C using 5 mM 2-AP (blue diamond) and 15 mM 2-AP (red diamond), and reaction at 40 °C using 5 mM 2-AP (green circle) and 15 mM 2-AP (purple circle). Resection times 30, 60, and 120 min were tested and the grafting yield was increased with increasing the reaction time. The solid and dashed lines are guides for general trend for the studied conditions.

In general, adding a hydrophilic compound that carries carboxylic, hydroxylic, or aminic groups will result in enhancing the hydrophilicity of the modified surface [60,61]. This phenomenon will be pronounced only in cases where these groups are kept free for bonding to water molecules [62]. As seen in Figure 4, pure water flux of the modified membranes was increased up to 15.4% relative to the blank membrane. This unexpected increase in the membrane flux may be interpreted to the formation of homogeneous layer without clogging the pores (see SEM images in the following discussion). In addition, the pore size of the membrane used in this study is relatively wide (0.2 µm), which can be considered a reason for diminishing any negative effect of modification on the original flux of the membrane. Meanwhile, the highest pure water flux reduction is not at the highest grafting yield but it was found to be 1.4% for the membrane modified using 15 mM 2-AP modifier, 120 min reaction time, and 25 °C reaction temperature. Generally, the PES membranes modified at 25 °C show comparable pure water flux to those membranes modified at 40 °C when using low modifier concentration (2-AP concentration). This is a logical result considering the limitation of low concentration (5 mM) to extend the reaction. However, when using 15 mM 2-AP concentration, there are plenty of monomers can be reacted with each other (homopolymer) as well as bonding onto the membrane surface. At 25 °C, the speed (i.e., the speed of colored homopolymers formation was noticed by the naked eyes) of formation a free radical is comparable to the rate of bonding this free radical onto the membrane surface and/or react with other free radicals inside the reaction medium (homopolymer formation) [63,64,65]. The overall speed of the reaction may be slow but the possibility of grafting is probably high [62,63], whereas, at 40 °C, the speed of free radical formation is high and presence of plenty of free radicals cause the acceleration of the reaction of monomers with each other (the homopolymer formation inside the reaction medium was noticeable by naked eye and the homopolymer formation process is faster at 40 °C than at 25 °C reaction temperature). The fast homopolymer formation may result in reducing the possibility of grafting on the membrane surface and increases the chance of adsorption/grafting of ready-formed oligomers on the membrane surface [61]. The general observation is that the added 2-AP modifier may be mainly grafted on the membrane surface at 25 °C reaction temperature, whereas it may be mainly adsorbed on and/or onto membrane surface at 40 °C reaction temperature especially in case of using high concentration of the modifier (15 mM 2-AP) [49,53]. This expected high reaction speed at 40 °C has been assisted by increase the solubility of the modifier at this temperature [61].

On the other hand, the increasing in the reaction temperature accelerates the overall reaction and the added 2-AP modifier per unit area of the membranes was doubled with the increase of the number of the added polar groups on the membrane surface and, consequently, the water flux was increased. In addition, (as shown in SEM of the membrane cross section modified at 40 °C reaction temperature), the added modifier may cause sticking of some fibers and creation more open cavities inside the membranes. Theses created open cavities can improve the pure water flux than the flux of the membranes modified at 25 °C [53].

In addition, we can observe similar trend for each studied condition: first the pure water flux was increased and then was decreased with increasing the grafting yield (although of the membrane fluxes are still higher than the flux of the blank membrane). This trend may be attributed to the decreases in the number of open (free) groups that have the ability to react with the water molecules and facilitate the water flux [53,66]. Thus, the low grafting yield may show better flux and better protein repellence than the high grafting yield [49]. Using wide pore size (0.2 µm) membranes minimized the effect of modification on the membranes original flux. Obviously, using much lower membrane pore size could cause clogging of the membrane pores that will severely affect the membrane original flux.

**Figure 5:**
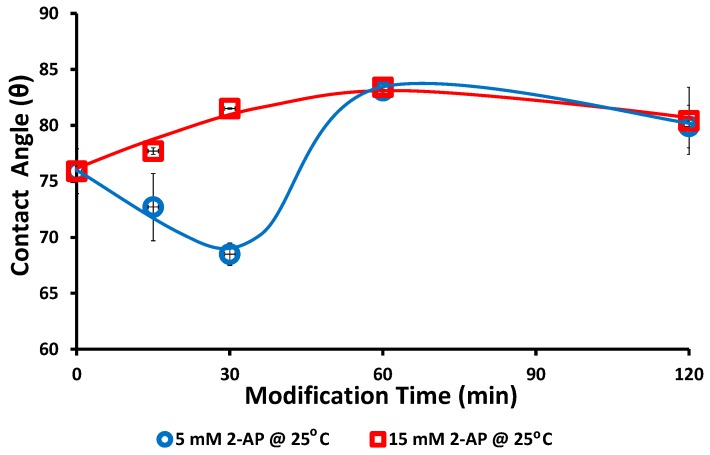
Change of the static water contact angle with the reaction (modification) time. Common reaction condition is pH 5.5 (0.1 M sodium acetate buffer), and 0.5 U·mL^−1^ enzyme (laccase). Two modifier concentrations, 5 mM (blue) and 15 mM (red) (2-aminophenol), were used at 25 °C reaction temperature and 15, 30, 60, and 120 min reaction times. The solid lines are guides for general trend for the studied conditions.

The static water contact angle was measured on the blank and the modified laminated PES layers to illustrate the effect of the modification on the hydrophobicity of the pure PES layer (surface). The effect of hydrophilic additives that were included in the used commercial membrane was avoided by using the laminated pure PES layer on the silica support. Different reaction times were used (15, 30, 60, and 120 min) at reaction temperature 25 °C, as shown in Figure 5.

Both low reaction time and low modifier concentration resulted in reduction in the static water contact angle (blue line in Figure 5), whereas carrying out the reaction for longer than 30 min resulted in increasing the static water contact angle (i.e., less hydrophilicity surface relative to the blank surface). This trend is in good agreement with the water flux of each studied set: first the flux was increased at low reaction time and then it was decreased at longer reaction time. In addition, the change in the static water contact angle assists our proposal that increasing the added materials is the reason for decreasing the water bounding sites on the modified membrane surface. Again, using high 2-AP modifier concentration resulted in gradually increasing the static water contact angle, as shown in Figure 5 (red line). Creation of wide cavities may result in the increasing of the water flux of the modified membrane, despite decreasing the surface hydrophilicity and increasing the static water contact angle, as observed in the membranes modified at 40 °C.

On the other hand, the structure of the produced oligomer or polymers can affect the properties of the produced modified membranes. The difference in the produced oligomers or polymers at different reaction (modification) conditions still needs further investigation using advanced chemical analyses techniques, which are underway.

**Figure 6:**
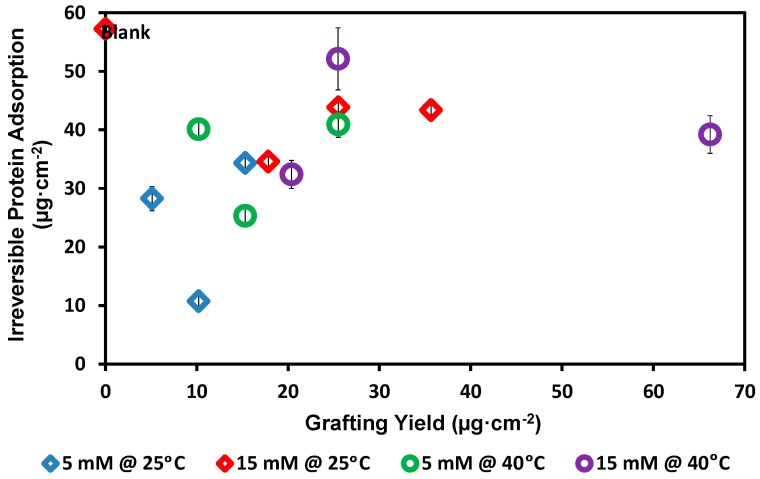
The effect of the grafting yield on the irreversible protein adsorption. Common reaction condition is pH 5.5 (0.1 M sodium acetate buffer), and 0.5 U·mL^−1^ enzyme (laccase). Reaction at 25 °C using 5 mM 2-AP (blue diamond) and 15 mM 2-AP (red diamond), and reaction at 40 °C using 5 mM 2-AP (green circle) and 15 mM 2-AP (purple circle). Resection times 30, 60, and 120 min were tested and the grafting yield was increased with increasing the reaction time. Adsorption test was done using 1 g·L^−1^ BSA, 0.1 M sodium acetate buffer (pH 7) and 25 °C.

The overall performance proved that the amount of bovine serum albumin (BSA) irreversibly adsorbed onto the blank membrane as shown in Figure 6, was decreased with modification. However, there is no straightforward relationship between the amount of added 2-AP modifier (the grafting yield) and the improvement of protein repellence of the modified membranes. It is well observed that the best reduction in protein adsorption was determined with the membranes modified using low 2-AP concentration (81.27% reduction in irreversible protein adsorption in case of using 5 mM 2-AP and 60 min modification reaction at 25 °C). We can see full agreement with the trend of the individual modification conditions that have been studied in this work (temperature, modifier concentration, etc.) regarding water flux and protein repellence. Grafting of 2-AP modifier on or onto PES membrane surface leads to an improvement in the protein repellence, but the excess of the grafting yield leads to slight decreases of both the membrane water flux and the protein repellence. In addition, the created wide cavities may work as pass rooms [54] for the protein, which increases the possibility of protein inclusion (molecules or aggregates) inside the membrane pores and consequently the irreversible protein adsorption was increased. Thus, keeping both low 2-AP modifier concentration and lower reaction temperature produce an effective modified membrane that shows good protein repellence. Clearly, in this study, all the modified membranes showed lower total irreversible adsorbed protein than the blank membranes.

**Figure 7:**
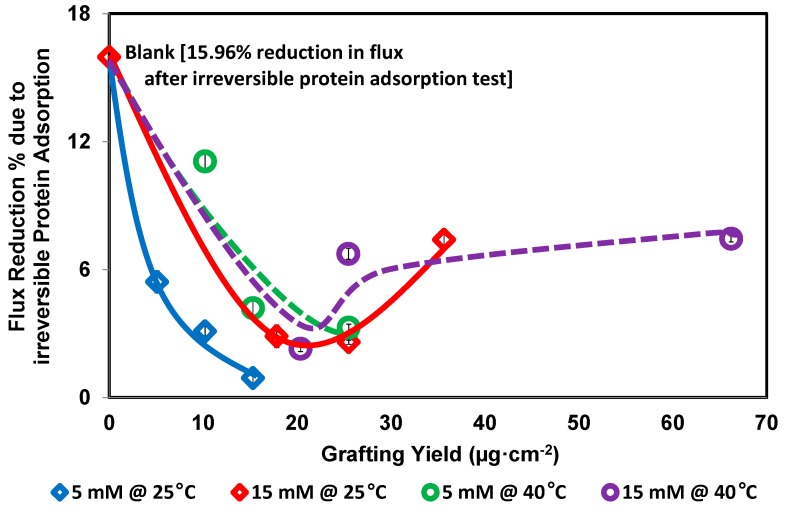
Flux reduction percent due to the irreversible protein adsorption at different grafting yield. Common reaction condition is pH 5.5 (0.1 M sodium acetate buffer), and 0.5 U·mL^−1^ enzyme (laccase). Reaction at 25 °C using 5 mM 2-AP (blue diamond) and 15 mM 2-AP (red diamond), and reaction at 40 °C using 5 mM 2-AP (green circle) and 15 mM 2-AP (purple circle). Resection times 30, 60, and 120 min were tested and the grafting yield was increased with increasing the reaction time. The solid and dashed lines are guides for general trend for the studied conditions.

The reduction in the water flux of the modified membranes due to the irreversible protein adsorption (un-removed protein after back and forward washing) is much lower than the reduction in the water flux of the blank membrane, as illustrated in Figure 7. The flux of the blank membrane was reduced by 15.96% of its original flux, whereas the modified membranes showed residual fluxes much better than the residual flux of the blank membrane. For example, the modified membrane that showed the best protein repellence lost 3.1% of its original flux due to modification. Thus, the flux of this modified membrane after both the modification and the irreversible protein adsorption test, is still higher than the flux of the blank membrane before protein adsorption by 11.81% and higher than the flux of the blank membrane after irreversible protein adsorption by 33.4% (i.e., fluxes due to irreversible protein adsorption were 11.74 and 15.62 m^3^·m^−2^·h^−1^ for the blank membrane and the modified membrane, respectively).

In addition, the residual flux of the modified membrane that acquired the highest grafting yield (66.21 µg·cm^−2^) is better than the flux of the blank membrane before and after irreversible protein adsorption (i.e., 13.97 and 11.74 m^3^·m^−2^·h^−1^ for the blank membrane, and 15.44 and 14.29 m^3^·m^−2^·h^−1^ for the modified membrane before and after irreversible protein adsorption, respectively). Thus, the residual flux of the modified membranes (after modification and the irreversible protein adsorption test) was much higher than the flux of the blank membrane.

**Figure 8:**
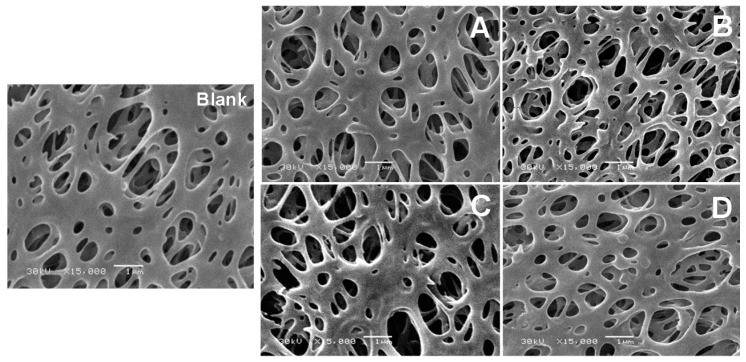
The scanning electron microscope (SEM) images for the blank poly(ethersulfone) (PES) membrane, and the modified PES membranes: (**A**,**B**) modified PES membranes using 5 and 15 mM modifier (2-AP), respectively at 25 °C; and (**C**,**D**) modified PES membranes using 5 and 15 mM 2-AP, respectively at 40 °C. Common reaction condition is pH 5.5 (0.1 M sodium acetate buffer), 0.5 U·mL^−1^ enzyme (laccase), and 120 min modification time. Magnification is 15,000×; and scale bar is 1 µm.

The SEM images of the blank PES membrane and the modified PES membranes for four different modification conditions are shown in Figure 8. The new layer formed on the top of the membrane surfaces is not easily noticeable on printed paper (clearly seen on the screen of SEM). The new layer seems thicker at modification temperature of 40 °C than at temperature 25 °C. In addition, using high modifier concentration (15 mM 2-AP) gives the chance for more clearly formed layer, especially at longer reaction (modification) time (120 min).

**Figure 9:**
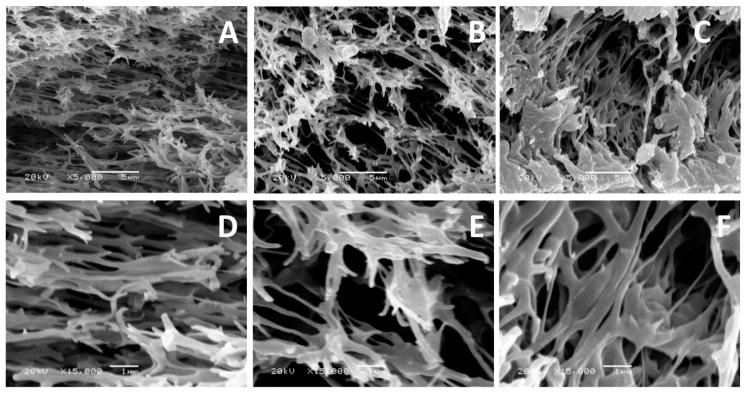
The scanning electron microscope (SEM) images for the cross-section of the blank poly(ethersulfone) (PES) membrane, (**A**) 5000× and (**D**) 15,000×; the modified PES membrane using 5 mM 2-AP and at 25 °C reaction temperature, (**B**) 5000× and (**E**) 15,000×; and modified PES membrane using 15 mM 2-AP and at 40 °C, (**C**) 5000× and (**F**) 15,000×. Common reaction condition is pH 5.5 (0.1 M sodium acetate buffer), 0.5 U·mL^−1^ enzyme (laccase), and 60 min reaction (modification) time.

No homopolymer lumps are shown in imaged PES surfaces in Figure 8. This may be attributed to using a modifier with two legs that tends to growth in one dimension with formation of a homogenous layer [49]. In addition, this may be related to the structure of formed layer that may consist of a repeating phenozaxine units as proposed in the previous electrochemical polymerization of 2-AP [41,42]. Moreover, the imaged cross-section, as shown in Figure 9C, illustrated that the effect of using higher 2-AP modifier concentration in the modification process takes place at higher reaction temperature (40 °C). The inside fibers seem not only thicker than those fibers modified at 25 °C but also they were stacked onto each other and new cavities inside the membranes have been created. The formed cavities may be the reason for enhanced membrane flux and reduced protein repellence.

**Figure 10:**
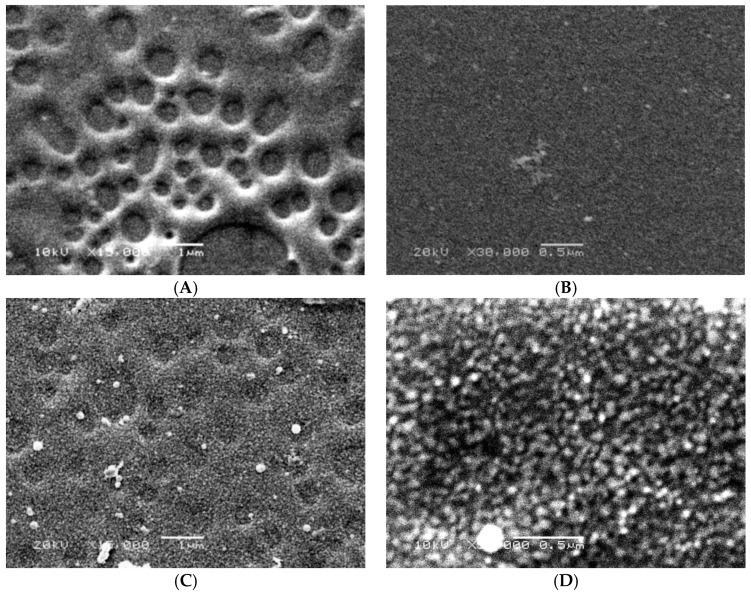
The scanning electron microscope (SEM) of the laminated poly(ethersulfone) (PES) layer on silicon dioxide slides using 15,000× and 30,000× magnification: (**A**,**B**) the blank laminated PES layer at the two magnifications; and (**C**,**D**) the modified laminated PES layer at the two magnifications using modification condition: 15 mM 2-AP, pH 5.5 (0.1 M sodium acetate buffer), and 0.5 U·mL^−1^ enzyme (laccase) modified for 60 min at 25 °C.

**Figure 11:**
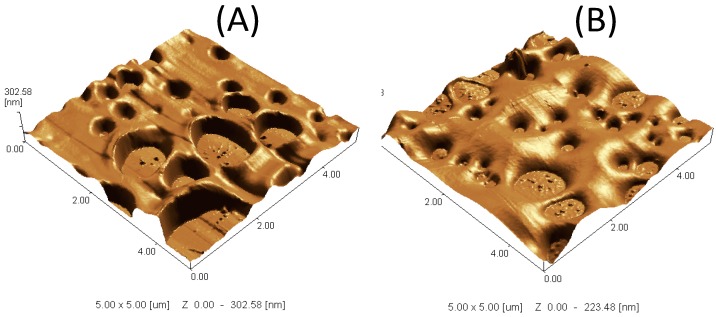
The scanning probe microscope (SPM) of the laminated PES layer on silicon dioxide slides: the blank laminated PES layer (**A**); and modified laminated PES layer (**B**). Modification condition: 15 mM 2-AP, pH 5.5 (0.1 M sodium acetate buffer), and 0.5 U·mL^−1^ enzyme (laccase) modified for 120 min at 25 °C.

To figure out the shape of the formed top layer, SEM imaging of the laminated PES layer on silicon dioxide slide was performed. As shown in Figure 10A, the blank PES layer appears as porous layer, which is analogous to the pores of the membrane. The modification resulted in oligomer chains growth on the surface, as shown in Figure 10C. To illustrate the bound poly(2-AP), the surfaces have been imaged with higher magnification, Figure 10B for the blank laminated PES layer and Figure 10D for the modified laminated PES layer. The grown tiny oligomers are noticeable all over the surface. Moreover, the formed layer of tiny oligomers was shown by SPM in Figure 11.

As shown in Figure 11, the added poly(2-AP) leads to the disappearance of the sharp edges of the pores of the blank layer. This is accompanied by the reduction in the layer roughness as illustrated in *Z* value, which was reduced from 302 to 233 nm. The formed layer appears as pancake shaped. Although the small pores may be decreased in size, they still open and for that the effect of modification in the membrane flux was minimum. The reader should keep in mind the difference between the used dense laminated PES layers (surfaces) and the commercial membranes to conclude that the added material on membranes has no effect on the pore size of the membranes, as shown in Figure 8.

**Figure 12:**
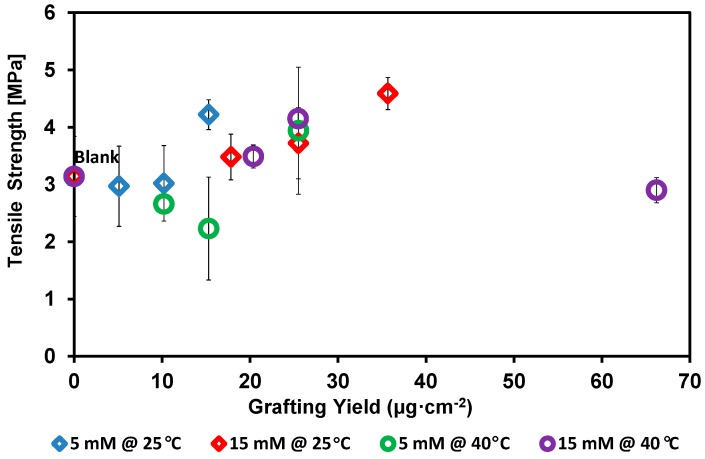
Effect of the grafting yield on the tensile strength of the blank poly(ethersulfone) (PES) membrane. Common reaction condition is pH 5.5 (0.1 M sodium acetate buffer), and 0.5 U·mL^−1^ enzyme (laccase). Reaction at 25 °C using 5 mM 2-AP (blue diamond) and 15 mM 2-AP (red diamond), and reaction at 40 °C using 5 mM 2-AP (green circle) and 15 mM 2-AP (purple circle). Resection times 30, 60, and 120 min were tested and the grafting yield was increased with increasing the reaction time.

The tensile strength of the blank and the modified PES membranes was measured to illustrate the effect of modification on the strength of the blank PES membrane. As shown in Figure 12, very slight reduction in the tensile strength of some modified membranes at low grafting yield was determined. This may be related to the bounding of the first oligomer. A very slight decrease in the membrane strength with modification at 40 °C is noticeable. However, in general, most of the modified membranes showed a comparable strength for the blank membrane. Actually, slight improvement in the strength of membranes modified at some modification conditions was determined.

**Figure 13:**
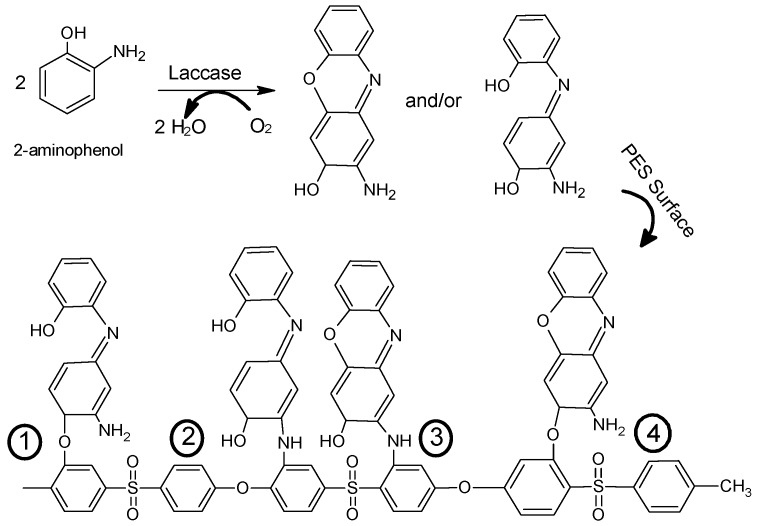
Schematic representation of four possible chemical structures of the poly(ethersulfone) (PES) surface after modification with 2-aminophenol, containing O-linked and N-linked structures.

There are four possible structures for the formed oligomers or polymers on the PES surface, as shown in Figure 13. The electrochemical polymerization of 2-AP has been determined in previous work as an oligomer of a ladder polymer structure with repeating phenozaxine units [41,42]. In addition, a linear dimer formed by N–N coupling: 2,2′-dihydroxyazobenzene has been identified as a polymerization product of 2-AP in non-acidic media [42]. The used pH is 5.5, for which a ladder polymer structure with repeating phenozaxine units is the most probable structure formed on the membrane surface. Further chemical analyses of the formed oligomers inside the reaction medium and of the formed modifying layers on the membrane surfaces are underway.

## 4. Conclusions

In this work, 2-AP was used to modify the PES membranes by using laccase biocatalyst. This modification resulted in a remarkable reduction in protein adsorption, whereas the water flux was increased. This modification does not harmfully affect the mechanical properties of the membranes. At different modification conditions, the formed modification layer seems homogenous all over the surface without formation of big lumps of homopolymers on the membrane surfaces, as shown in previous work using 4-hydroxybenzoic acid modifier [49]. The structure of the formed modifying layer(s) is under investigation using other advanced chemical analytical techniques.
